# CXCL10 is a potential biomarker and associated with immune infiltration in human papillary thyroid cancer

**DOI:** 10.1042/BSR20203459

**Published:** 2021-01-07

**Authors:** Xiao-Jing Qin, Xu Lin, Gang Xue, Hui-Li Fan, Hao-Yu Wang, Jing-Fang Wu, Da Pei

**Affiliations:** 1 Department of Histology and Embryology, Hebei North University, Zhangjiakou 075000, China; 2 Department of Otorhinolaryngology Head and Neck Surgery, Hebei North University, Zhangjiakou 075000, China

**Keywords:** bioinformatics, cancer, tumor

## Abstract

Background: In recent years, the annual incidence of thyroid cancer (TC) has increased, with papillary thyroid cancer (PTC) identified as the most commonwinwordpathological type accounting for approximately 80% of all thyroid cancer cases. The tumor microenvironment is known to play a vital role in tumor information transmission and immune detection.

Methods: In the present study, we examined gene expression data from 518 patients with PTC. The ESTIMATE algorithm was used to calculate immune and stromal scores of PTC patients. Based on a protein–protein interaction (PPI) network, functional enrichment and overall survival analyses, C-X-C motif chemokine ligand 10 (CXCL10) was identified as a core gene. We further investigated the roles of core genes of PTC in the tumor immune microenvironment using LinkedOmics, GSEA, and TIMER tools.

Results: Immune, stromal and ESTIMATE scores were related to clinicopathological variables of patients with PTC, but not survival outcomes. Eight differentially expressed genes (DEGs) were associated with survival outcome. In addition, immunochemical staining experiments revealed lower expression of CXCL10 in PTC than paracancerous tissues. GSEA pathway enrichment analysis revealed downregulation of CXCL10 in multiple cancer pathways. CXCL10 and related genes were enriched in pathways related to adaptive immune response, cellular defense response and regulation of innate immune response.

Conclusion: The tumor microenvironment plays a critical role in development of PTC and CXCL10 may serve as a novel target of precision therapy for this patient population.

## Introduction

Thyroid cancer is not only the most common malignancy of the human endocrine system, accounting for ∼3% of the total incidence of systemic malignant tumors, but also one of the fastest growing malignant solid tumors. According to the histopathological classification system, thyroid cancer is mainly categorized into papillary thyroid, follicular thyroid, medullary thyroid and undifferentiated thyroid cancer types. Among these, papillary thyroid carcinoma accounts for approximately 80% of all pathological thyroid cancer cases [1]. In 2018, approximately 52,070 new cases of thyroid cancer in the United States were recorded [2]. Due to improvements in diagnostic techniques, such as ultrasound and fine-needle biopsy, the incidence of PTC has been increasingly reported in recent years [3]. The majority of PTC patients have slow disease progression and good prognosis, with a 10-year survival rate of more than 90%. However, patients with papillary thyroid microcarcinoma commonly develop lymph node metastasis at an early stage of disease with an incidence of 30–40%, leading to a 50% decrease in the 10-year survival rate [4–6]. Lymph node metastasis from thyroid cancer cells serves as an independent risk factor for poor prognosis of PTC, with more than 8–10% eventually evolving to distant metastasis or potential cancer-related death [7–10]. Early and effective interventions are critical for diagnosis of PTC and blocking the development of lymph node metastasis in thyroid cancer cells, which would significantly improve patient outcomes. Therefore, identification of biomarkers related to the tumor microenvironment is currently an important consideration in the clinical management of PTC.

Recent studies suggest that immune cells, such as macrophages, mast cells, neutrophils, and lymphocytes, play a tumorigenic role in PTC, supporting the potential efficacy of targeted immunotherapy as a treatment strategy [11]. Immune escape, an important feature of tumor malignancy, plays a critical regulatory role in the evolution of many solid tumor types. The term ‘tumor microenvironment’ is used to describe the milieu surrounding tumor cells composed of inflammatory mediators, extracellular stromal molecules, and immune cells [12]. Essential non-tumor components of the tumor microenvironment are immune and stromal cells, which are critical for transmitting information and stimulating the formation and evolution of tumors. The diagnostic and prognostic value of immune and stromal cells in tumors has been established [13,14]. Tumor immune escape can be modulated by immune cell components in the microenvironment, where by alterations in these components promote the formation of an immunosuppressive state [15]. Clinicopathological typing of thyroid cancer patients is reported to be closely related to immune and inflammatory cells in the tumor microenvironment [16,17]. The ESTIMATE algorithm has been further used to evaluate immune and stromal scores through analysis of expression patterns of specific genes in immune and stromal cells for the purpose of predicting cancer cell infiltration into non-tumor cells [18]. The ESTIMATE algorithm has been increasingly applied to a variety of tumor models, including breast and colon cancer types [19,20]. However, to our knowledge, a few studies have explored the ratio of stromal and immune cells in PTC using the ESTIMATE algorithm.

In the present study, we evaluated immune scores by extracting tumor microenvironment-related genes based on TCGA data of PTC patients and the ESTIMATE algorithm and explored one or several gene modules playing crucial roles in the PTC microenvironment, with a view to providing a theoretical basis for further research.

## Materials and methods

### TCGA database

Gene expression data for thyroid cancer patients were retrieved from the TCGA database (https://tcga-data.nci.nih.gov/tcga/). The RNA expression profiles of PTC patients were further analyzed and annotation data of the Affymetrix HT human genome U133 array plate set extracted. Clinical information, including gender, age, histologic grade, pathologic stage, survival and outcome, were additionally extracted from TCGA. After downloading the data, the ESTIMATE algorithm was implemented in the form of the ‘ESTIMATE’ package in R for calculation of the immune and stromal scores [18].

### Identification of differentially expressed genes

To improve screening for differentially expressed genes, the limma software package was employed [21]. Differentially expressed genes (DEGs) were identified based on cutoffs of fold change > 2 and adj. *P*<0.05.

### Heatmaps and clustering analysis

To construct cluster analysis and immune stromal heatmaps, we used pheatmap R software package [22].

### Construction of the PPI network

To analyze protein–protein interactions (PPI), the STRING database was employed [23]. To clarify the interactions between genes, protein–protein interaction networks of genes that were up- or down-regulated were constructed based on immune and stromal scores and reconstructed via Cytoscape software. The active interaction source settings were as follows: text mining, experiment, database, co-expression, neighborhood, gene fusion and co-occurrence. The minimum interaction score requirement was 0.4. Only a single network consisting of more than 10 nodes was shown and allowed to proceed to the next level of analysis. The MCODE plug in was subsequently used to analyze closely related gene modules.

### Overall survival analysis

To evaluate the prognostic value of DEGs in thyroid cancer, Kaplan–Meier survival analysis was used and overall survival curves obtained using Kaplan–Meier plotter.

### Enrichment analysis of DEGs

To establish the potential biological functions of intersecting DEGs, KEGG pathway enrichment [24] and GO [25] analyses were performed using the Profiler package. GO assessed biological processes (BP), molecular functions (MF) and cellular components (CC). The results from both GO and KEGG pathway analyses were processed using ‘clusterProfiler’, ‘enrichplot’ and ‘ggplot2’ packages. The results were visualized with the ggplot2 package. The *P* value cut-off was set at 0.05.

### LinkedOmics

The LinkedOmics (http://www.linkedomics.org/) database is a unique platform for accessing, analyzing and comparing multi-omics data within and between tumor types, providing a convenient resource for biologists and clinicians [26]. To explore the kinase target of the PTC hub gene, CXCL10, the LinkInterpreter module of LinkedOmics was applied in the present study.

### TIMER

The TIMER (https://cistrome.shinyapps.io/timer/) database is a practical platform for systematic evaluation of the interactions between tumor and immune cells [27]. In the current study, we aimed to explore the associations among immune infiltrates, immunomodulatory factors and CXCL10 gene expression. All analyses were based on the TCGA TCHA dataset (*N*= 501).

### Open targets

Open targets provide a target-centric workflow to aid in identifying diseases potentially related to specific targets [28]. Here, we used Open targets to explore diseases related to CXCL10.

### GeneMANIA

GeneMANIA (www.genemania.org) is a portal to establish protein–protein interaction (PPI) networks and provide insights into the functions of submitted genes [29]. In the present study, GeneMANIA was used to visualize gene networks of CXCL10 and its related genes.

### GSCALite

GSCALite (http://bioinfo.life.hust.edu.cn/web/GSCALite/) is a portal website providing a platform for analysis of gene sets in cancer. Genomic aberrations can affect clinical response to treatment and serve as potential biomarkers for drug screening [30]. We integrated 265 small molecules from Cancer Drug Sensitivity Genomics (GDSC) and analyzed the expression of each gene in the genome in relation to small molecule/drug sensitivity (IC_50_) via Spearman correlation analysis. *P*-values less than 0.05 were considered statistically significant.

### Gene set enrichment analysis

GSEA is a calculation method that performs genome-wide expression profiling of two sample types, with the aim of identifying significant and consistent differences. In this study, according to CXCL10 expression patterns, samples of PTC were divided into high and low expression groups. GSEA was subsequently applied to evaluate the differences between the high and low CXCL10 expression groups. Data were considered significant at *P*<0.05 and the false discovery rate (FDR) was <25%. According to the normalized enrichment score (NES), an enrichment approach related to the biological process of papillary thyroid carcinoma was selected.

### Immunohistochemical (IHC) staining and evaluation

For IHC, PTC tissue microarrays were firstly deparaffinized with xylene and rehydrated with a gradient of ethanol to distilled water. Antigen retrieval was performed by heating the tissue microarrays in 10 mM sodium citrate buffer for 30 min. Next, endogenous peroxidase was blocked with 3% H_2_O_2_ and the microarray treated with normal goat serum working solution for 30 min to reduce non-specific binding. After overnight incubation with the rabbit monoclonal anti-CXCL10 antibody (1:100 dilution, BOSTER, Wu Han, China, BA4723) at 4°C, 120-point PTC tissue microarrays were washed with PBS and incubated with broad-spectrum secondary antibodies. Slides were further washed with PBS, followed by incubation with horseradish enzyme-labeled streptavidin working solution at 37°C for 30 minutes. After a further three washes with PBS, signal detection was performed with the DAB staining kit. According to Remmele's semi-quantitative immune response score (IRS) scale, the overall IHC score (1–5) was evaluated [31].

## Results

### Patient characteristics

**Figure 1:**
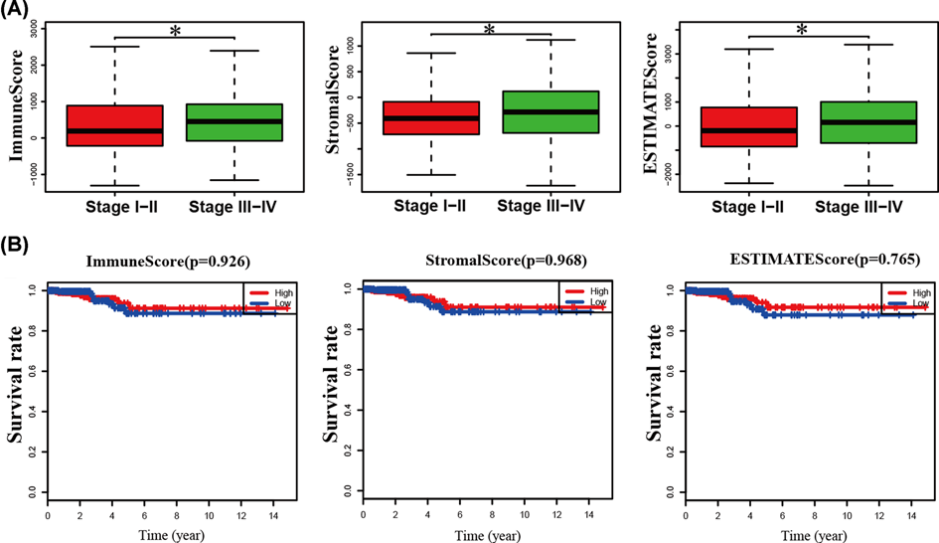
Correlations among immune/stromal score, clinical pathological variables and overall survival in PTC (**A**) Analysis of tumor stage based on immune score (*P*=0.044), stromal score (*P*=0.048) and ESTIMATE score (*P*=0.046). (**B**) No significant differences in PTC overall survival analysis were evident based on immune/matrix/ESTIMATE score; *, P<0.05.

In March 2020, the gene expression profiles and corresponding clinicopathological data from 518 PTC patients, including 369 (70.89%) female patients and 149 (29.11%) male patients, were extracted from the TCGA database. The minimum age of initial diagnosis was 15 years and average age was 46 years. PTC stages were distributed as follows: I (55.98%, *n*=290), II (11%, *n*=57), III (23.16%, *n*=120) and IV (9.84%, *n*=51). Using the ESTIMATE method, immune scores were in the range of -1304.72 to 3233.39, stromal scores were -1714.40 to 1608.17and ESTIMATE scores were -2470.47 to 4199.50. With increase in the tumor staging level, the corresponding average immune (*P*=0.044), stromal and ESTIMATE (*P*=0.046) scores were increased (Figure 1). Next, Kaplan–Meier survival analysis was conducted to establish the associations of immune, stromal and ESTIMATE scores with prognosis. Patients with PTC were divided into high and low score groups. However, none of the scores were associated with survival outcomes in PTC (Figure 1).

### Gene expression profiles associated with immune scores and stromal scores in PTC

**Figure 2:**
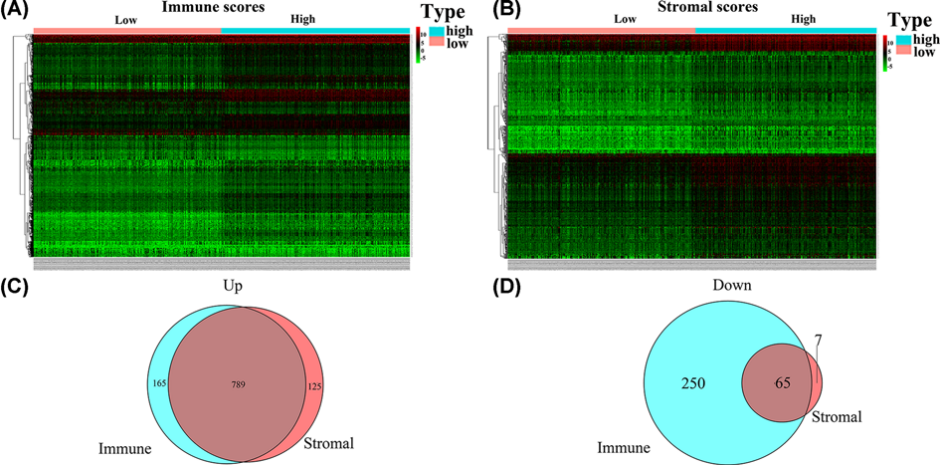
Relationship between gene expression profiles and immune/stromal scores in PTC (**A** and **B**) Heatmap showing significant DEGs based on immune and stromal scores. Red represents higher expression and green represents lower expression. Venn diagram showing the number of DEGs up- (**C**) or down-regulated (**D**) in the immune and stromal score groups.

To obtain the gene expression profiles of patients with PTC in association with immune and stromal scores, Affymetrix microarray data analysis was conducted on 518 PTC samples from patients in the TCGA database. The population with a higher immune score, presented in heatmap2A, had a total of 1269 DEGs, including 954 up-regulated and 315 down-regulated genes. Based on the higher stromal score (Figure 2B), 986 DEGs were identified, including 914 up-regulated and 72 down-regulated genes. Venn diagram analysis disclosed that compared with the low group, the high group had a total of 854 DEGs, including 789 up-regulated and 65 down-regulated genes (Figure 2C,D).

### Functional enrichment analysis of DEGs

**Figure 3:**
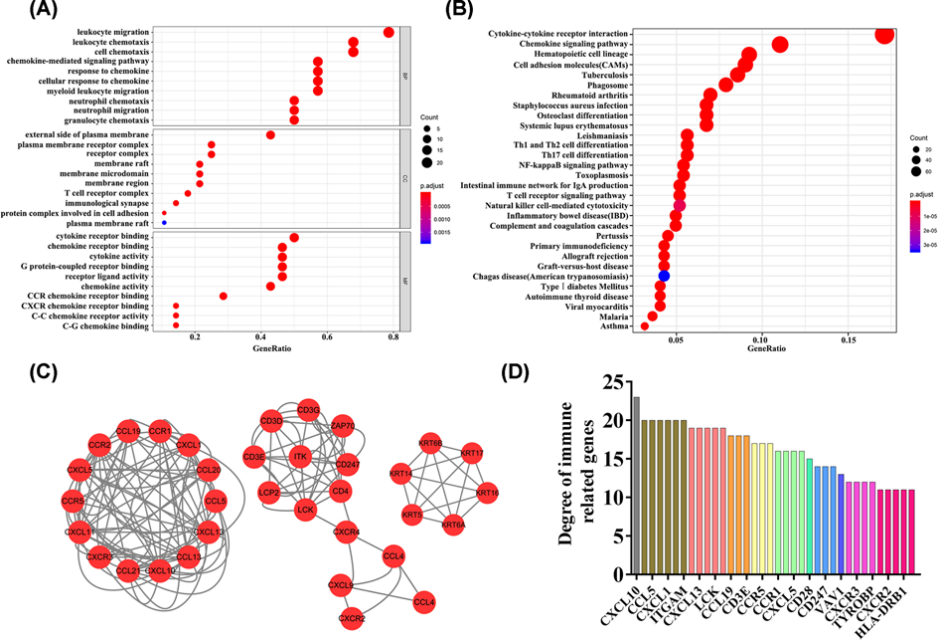
Functional enrichment analysis of DEGs (**A**) Top 10 terms in GO analysis (*P*<0.05). (**B**) Enriched terms in KEGG pathway analysis (*P*<0.05). (**C**) Top three PPI networks of CXCL10, KRT, and ZAP70 modules. The network includes three modules and 14 nodes showing the association between tumor microenvironment-related genes and PTC. (**D**) Bar chart signifying important genes (degree>10) in the PPI network.

To establish the functions of DEGs, enrichment analysis using GO and KEGG functions was conducted. GO analysis demonstrated that the main ‘Biological Process’ (BP) ontology was enriched in leukocyte migration, leukocyte chemotaxis, cell chemotaxis, chemokine-mediated signaling pathway, response to chemokine, cellular response to chemokine, myeloid leukocyte migration, neutrophil chemotaxis, neutrophil migration and granulocyte chemotaxis functions. For the ‘Cellular Component’ (CC) ontology, enriched terms included the external side of the plasma membrane, plasma membrane receptor complex, receptor complex, membrane raft, membrane microdomain, membrane region, T-cell receptor complex, immunological synapse, protein complex involved in cell adhesion and plasma membrane raft. With regard to ‘Molecular Function’ (MF), cytokine receptor binding, chemokine receptor binding, cytokine activity, G protein-coupled receptor binding, receptor ligand activity, chemokine activity, CCR chemokine receptor binding, CXCR chemokine receptor binding, C-C chemokine receptor activity and C-G chemokine binding terms were enriched (Figure 3A). KEGG pathway analysis additionally disclosed involvement of DEGs in cytokine–cytokine receptor interactions and the chemokine signaling pathway (Figure 3B). PPI network analysis was an important analytical tool to identify the critical genes associated with PTC interactions from a systems perspective. In the present study, the top three PPI networks of CXCL10, KRT, and ZAP70 modules were selected using Cytoscape software (Figure 3C).Greater connectivity was signified by larger gene nodes. The top five connected genes were CXCL10, CCL20, CCL5, CD4, and CXCL1 (Figure 3D).

### Roles of individual DEGs in overall survival in PTC

**Figure 4:**
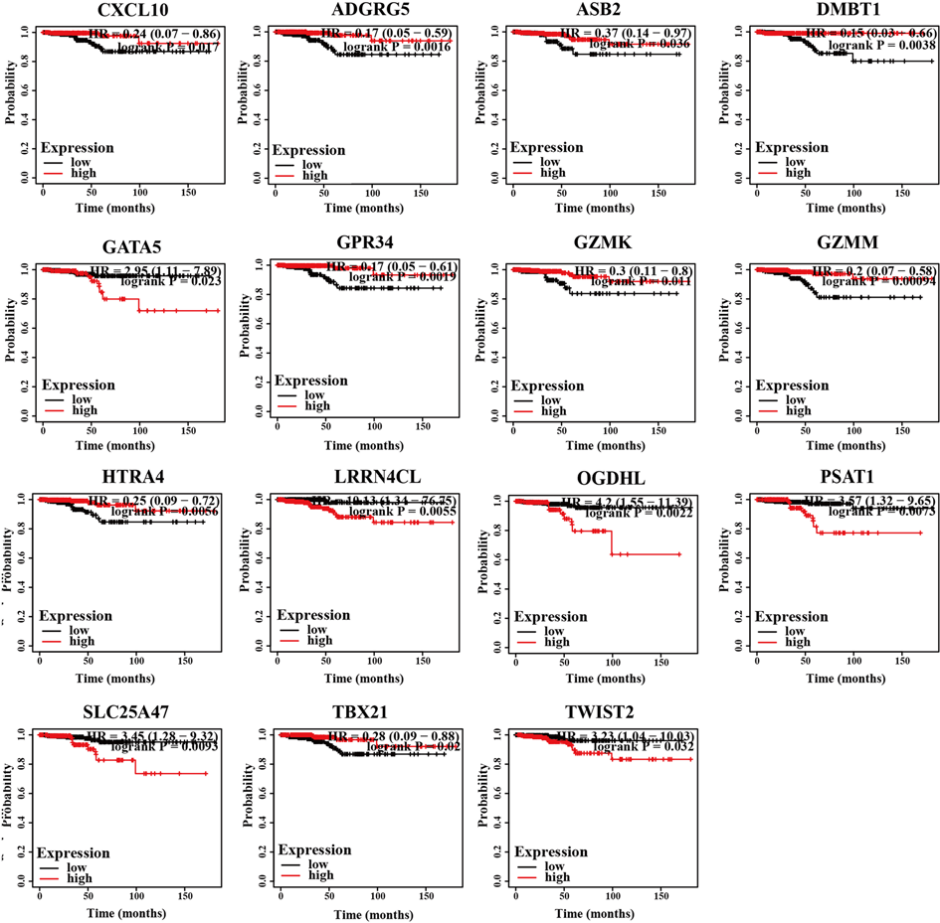
Kaplan–Meier analysis of the relationship between prognosis and DEGs related to the tumor microenvironment

To establish the relationships between the 789 up-regulated and 65 down-regulated genes and prognostic survival of PTC patients, Kaplan–Meier survival analysis was conducted. All TC tumor samples were divided into high and low expression groups based on specific genes. Overall, 15 genes were associated with prognosis and survival. Groups with high expression of GATA5 (HR = 2.95, 95% CI: 1.11–7.89, *P*=0.023), LRRN4CL (HR = 10.13, 95% CI: 1.34–76.75, *P*=0.0055), OGDHL (HR = 4.2, 95% CI: 1.55–11.93, *P*=0.0022), PSAT1 (HR = 3.57, 95% CI: 1.32–9.65, *P*=0.0073), SLC25A47 (HR = 3.45, 95% CI: 1.28–9.32, *P*=0.0093) and TWIST2 (HR = 3.23, 95% CI: 1.04–10.03 and *P*=0.032) showed poorer overall survival rates while higher survival rates were associated with high expression of CXCL10 (HR = 0.24, 95% CI: 0.07–0.86, *P*=0.017), ADGRG5 (HR = 0.17, 95% CI: 0.05–0.59, *P*=0.0016), ASB2 (HR = 0.37, 95% CI: 0.14–0.97, *P*=0.036), DMBT1 (HR = 0.15, 95% CI: 0.03–0.66, *P*=0.0038), GPR34 (HR = 0.17, 95% CI: 0.05–0.61, *P*=0.0019), GZMK (HR = 0.3, 95% CI: 0.11–0.8, *P*=0.011), GZMM (HR = 0.2, 95% CI: 0.07–0.58, *P*=0.00094), HTRA1 (HR = 0.25, 95% CI: 0.09–0.72, *P*=0.0056) and TBX21 (HR = 0.28, 95% CI: 0.09–0.88, and *P*=0.02; Figure 4).Accordingly, we speculate that expression levels of these 15 genes are critical for prognosis of PTC patients. Based on the highest degree, CXCL10 was selected as the core gene in the PTC immune microenvironment for further exploration.

### GO and KEGG pathway analyses of genes correlated with CXCL10 expression in PTC

**Figure 5:**
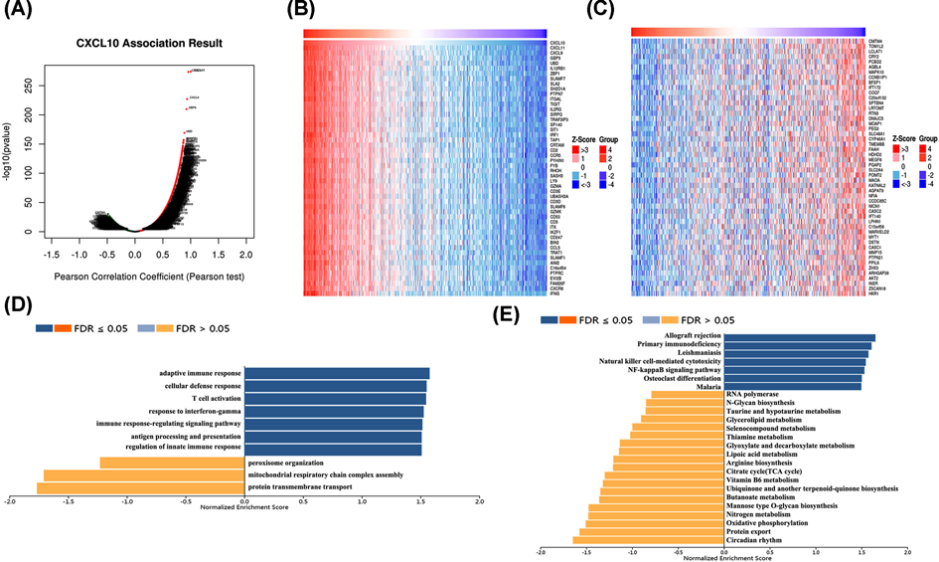
Co-expressed genes and functional enrichment analysis of CXCL10 (LinkedOmics) (**A**) Correlations between CXCL10 and differentially expressed genes in PTC. (**B** and** C**) Heatmaps indicate the top 50 genes positively and negatively correlated with CXCL10 in PTC. Red refers to positively correlated genes and green refers to negatively correlated genes. Statistical analysis was performed using Pearson’s test. GO function (**D**) and KEGG pathway (**E**) analyses of CXCL10.

**Figure 6:**
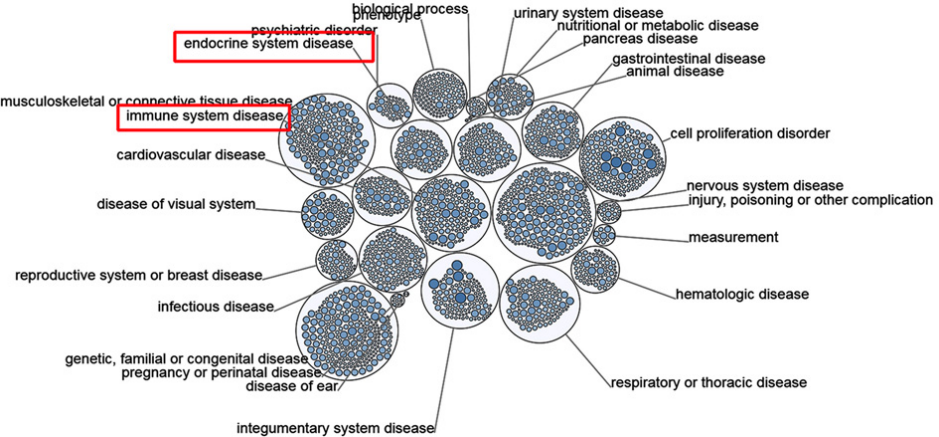
Diseases related to CXCL10 (Open Targets)

The genes related to CXCL10 and differentially expressed in PTC were investigated via LinkedOmics to establish the specific mechanisms of action of CXCL10 in thyroid cancer. As a result, 19,928 genes related to CXCL10 were identified. Among these, 10,157 genes (dark red dots) were positively correlated and 9769 genes (dark green dots) were negatively correlated with CXCL10 (Figure 5A; false discovery rate <0.05). The top 50 genes that were significantly positively and negatively correlated with CXCL10 are presented in Figure 5B,C, respectively. As shown in Figure 5D, CXCL10 and neighboring genes were mainly implicated in adaptive immune response, cellular defense response, T-cell activation, response to interferon-gamma, immune response, regulation of signaling pathways, antigen processing and presentation, and regulation of the innate immune response. The results of KEGG pathway analysis showed that the functions of CXCL10 and its neighboring genes were mainly enriched in the NF-kappaB signaling pathway, allograft rejection, primary immunodeficiency and leishmaniasis (Figure 5E). To further establish the diseases caused by aberrant expression of the CXCL10 gene, an analysis using the Open Targets website was conducted. As expected, CXCL10 was significantly implicated in diseases of the immune and endocrine systems (Figure 6). Our findings demonstrate that the CXCL10 gene plays an important regulatory role in immune pathways of the PTC microenvironment, supporting its utility as a novel immunotherapeutic target for PTC. The potential mechanisms of action of CXCL10 were further explored.

### Use of GSEA to identifyCXCL10-related signaling pathways

**Figure 7:**
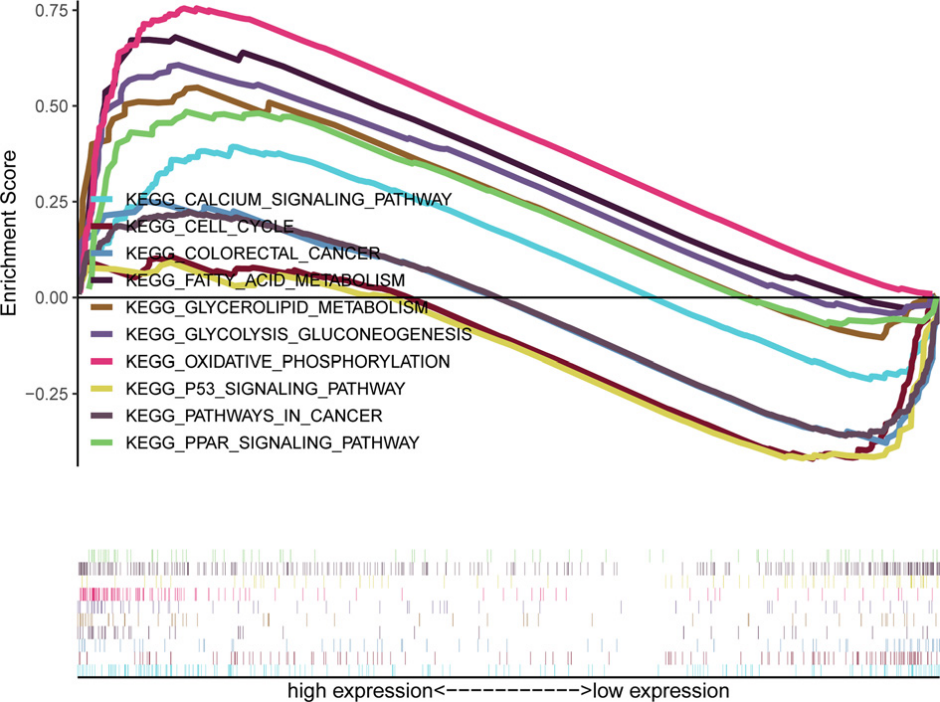
Gene set enrichment analysis (GSEA) of CXCL10 gene expression

To determine the CXCL10-associated signaling pathways involved in PTC, we conducted gene set enrichment analysis (GSEA) to determine differences in CXCL10 gene expression. The most significantly enriched signal pathway was identified based on the normalized enrichment scores (NES). High expression of CXCL10 was markedly associated with activation of oxidative phosphorylation, glycolysis gluconeogenesis, glycerolipid metabolism, PPAR signaling, fatty acid metabolism and calcium signaling pathways and inhibition of p53 signaling, cell cycle, pathways in cancer, and colorectal cancer signal pathways (Figure 7).

### Identification of the kinase target of CXCL10

**Figure 8:**
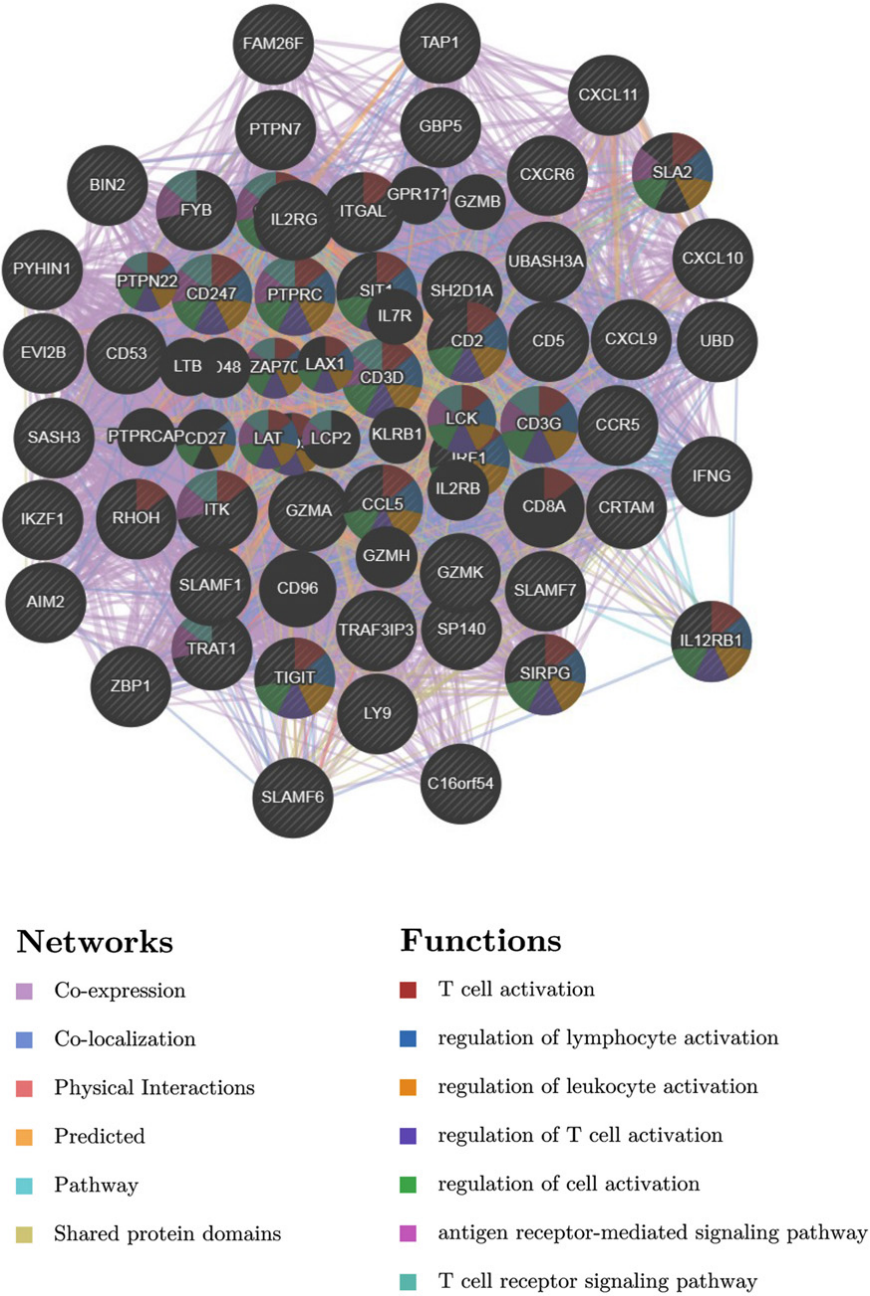
PPI networks of CXCL10 and related genes (GeneMANIA)

**Figure 9:**
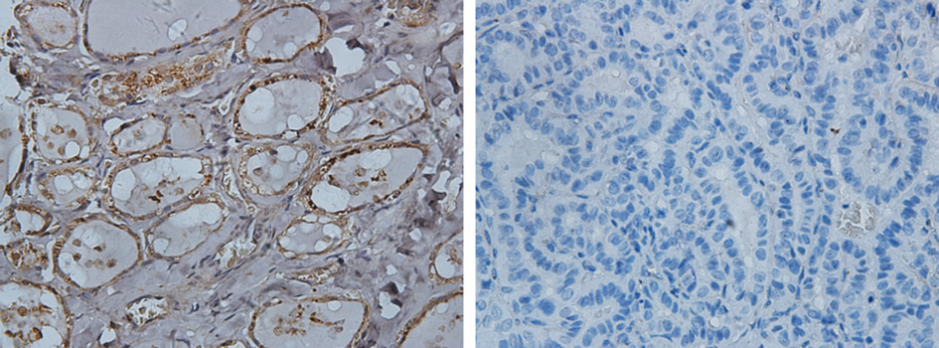
Expression of CXCL10 in PTC and tumor-adjacent tissues (CXCL10 protein levels were higher in tumor-adjacent tissues compared to PTC tissues; 400× magnification).

The potential kinase targets of CXCL10 in thyroid carcinoma were analyzed using the LinkedOmics database. The kinase network corresponding to CXCL10 included LCK, LYN, HCK and SYK (Table 1). To clarify the potential regulatory mechanisms between these four kinases and CXCL10, PPIs were constructed using GeneMANIA to establish their specific functions. The gene sets were mainly implicated in regulation of lymphocyte activation, regulation of leukocyte activation, regulation of T-cell activation, regulation of cell activation, antigen receptor-mediated signaling and T-cell receptor signaling pathways (Figure 8). Next, we performed tissue microarray immunohistochemical staining to evaluate expression of CXCL10 in PTC and matched adjacent tissues. Compared with adjacent tissues, expression of CXCL10 in PTC tissues was clearly decreased (Figure 9).

**Table 1:** The kinase target networks of CXCL10 in PTC (LinkedOmics)

	Enriched kinase target	Description	Leading Edge Num	*P* value
CXCL10	Kinase_LCK	LCKproto-oncogene, Srcfamily tyrosine kinase	22	0
	Kinase_LYN	LYN proto-oncogene, Src family tyrosine kinase	22	0
	Kinase_HCK	HCK proto-oncogene, Src family tyrosine kinase	8	0
	Kinase_SYK	spleen associated tyrosine kinase	15	0.0020704

### Correlation between CXCL10 and immune cell infiltration

**Figure 10:**
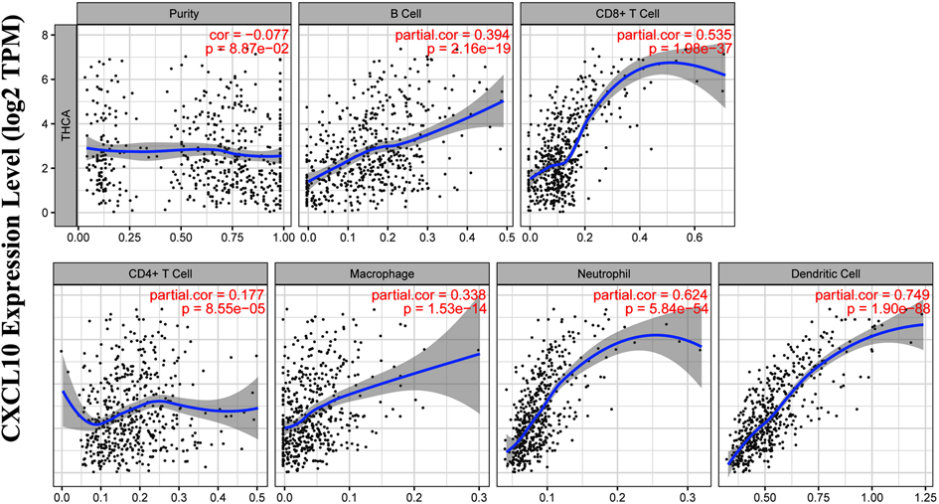
Relationship between CXCL10 and various infiltrating immune cells (TIMER)

We further analyzed the correlation between CXCL10 and immune cells infiltrating the tumor microenvironment using the TIMER database. CXCL10 expression was positively associated with B cell infiltration (Cor = 0.394, *P* = 2.16e-19), CD8 + T cells (Cor = 0.535, *P* = 1.98e-37), CD4 + T cells (Cor = 0.177, *P* = 8.55-05), macrophages (Cor = 0.338, *P* = 1.53e-14), neutrophils (Cor = 0.624, *P* = 5.84e-54) and dendritic cells (Cor = 0.749, *P* = 1.90e-88; Figure 10). The relationship between CXCL10 expression and a number of immune markers was further evaluated. As shown in Table 2, significant correlations (correlation coefficient> 0.6) were observed between CXCL10 and expression of PD-1 (PDCD1), CD2, CD19, CD79A, CD68, IL10, VSIG4, HLA-DPB1, CD11c (ITGAX), T-bet (TBX21), STAT1, IFN-g (IFNG), FOXP3 and CTLA4.

**Table 2:** Correlation analysis between CXCL10 and gene biomarkers of immune cells in THCA (TIMER)

Immune cells	Biomarkers	None		Purity	
		Cor	*P*-value	Cor	*P*-value
CD8+ T cell	CD8A	0.717	0.00E+00	0.725	3.91E-80
	CD8B	0.638	0.00E+00	0.643	2.46E-58
T cell (general)	CD3D	0.834	0.00E+00	0.837	3.96E-129
	CD3E	0.838	0.00E+00	0.843	4.90E-132
	CD2	0.846	1.15E-140	0.847	2.15E-135
B cell	CD19	0.684	1.71E-71	0.686	3.27E-69
	CD79A	0.767	1.24E-99	0.769	1.67E-96
Monocyte	CD86	0.796	0.00E+00	0.792	2.08E-106
	CD115(CSF1R)	0.703	0.00E+00	0.707	2.79E-75
TAM	CCL2	0.548	0.00E+00	0.541	1.92E-38
	CD68	0.698	1.10E-75	0.692	6.78E-71
	IL10	0.634	1.10E-58	0.632	1.13E-55
M1 Macrophage	INOS (NOS2)	0.101	2.30E-02	0.101	2.58E-02
	IRF5	0.489	5.18E-32	0.486	2.48E-30
	COX2(PTGS2)	0.337	6.84E-15	0.325	1.68E-13
M2 Macrophage	CD163	0.585	4.89E-48	0.577	1.23E-44
	VSIG4	0.654	1.61E-63	0.654	7.96E-61
	MS4A4A	0.701	0.00E+00	0.699	9.50E-73
Neutrophils	CD66b (CEACAM8)	0.187	2.24E-05	0.183	4.65E-05
	CD11b (ITGAM)	0.706	4.1E-76	-0.095	3.56E-02
	CCR7	0.741	0.00E+00	0.74	6.70E-86
Natural killer cell	KIR2DL1	0.243	2.95E-08	0.266	2.34E-09
	KIR2DL3	0.377	1.24E-18	0.37	2.78E-17
	KIR2DL4	0.422	1.92E-23	0.409	4.57E-21
	KIR3DL1	0.361	4.45E-17	0.367	5.49E-17
	KIR3DL2	0.524	2.56E-37	0.525	5.49E-36
	KIR3DL3	0.34	3.16E-15	0.337	1.92E-14
	KIR2DS4	0.305	2.06E-12	0.312	1.84E-12
Dendritic cell	HLA-DPB1	0.802	0.00E+00	0.801	2.86E-110
	HLA-DQB1	0.531	0.00E+00	0.528	2.03E-36
	HLA-DRA	0.795	0.00E+00	0.791	7.65E-108
	HLA-DPA1	0.797	0.00E+00	0.79	3.68E-105
	BDCA-1(CD1C)	0.506	1.94E-34	0.496	1.14E-31
	BDCA-4(NRP1)	0.04	3.63E-01	0.023	6.13E-01
	CD11c (ITGAX)	0.679	0.00E+00	0.671	4.52E-65
Th1	T-bet (TBX21)	0.722	3.28E-83	0.724	1.60E-80
	STAT4	0.598	0.00E+00	0.593	1.10E-47
	STAT1	0.698	1.86E-75	0.692	9.45E-71
	IFN-g (IFNG)	0.802	1.72E-115	0.803	2.39E-111
	TNF-a (TNF)	0.516	4.86E-36	0.51	1.17E-33
Th2	GATA3	0.211	1.48E-06	0.198	1.03E-05
	STAT6	0.118	7.62E-03	0.105	1.98E-02
	STAT5A	0.33	2.17E-14	0.329	8.56E-14
	IL13	0.196	8.37E-06	0.201	8.06E-06
Tfh	BCL6	0.162	2.38E-04	0.142	1.62E-03
Th17	STAT3	0.213	1.36E-06	0.195	1.49E-05
	IL17A	0.314	4.04E-13	0.318	6.11E-13
Treg	FOXP3	0.696	0.00E+00	0.703	4.75E-74
	CCR8	0.556	1.14E-42	0.548	1.62E-39
	STAT5B	-0.038	3.96E-01	-0.054	2.37E-01
	TGFb (TGFB1)	0.213	1.28E-06	0.205	5.05E-06
T-cell exhaustion	PD-1 (PDCD1)	0.678	1.08E-69	0.69	3.45E-70
	CTLA4	0.807	7.24E-118	0.804	5.16E-112
	LAG3	0.819	0.00E+00	0.828	2.22E-124
	TIM-3 (HAVCR2)	0.784	0.00E+00	0.78	6.40E-101
	GZMB	0.73	0.00E+00	0.737	8.01E-85

### Drug sensitivity analysis of hub genes

**Figure 11:**
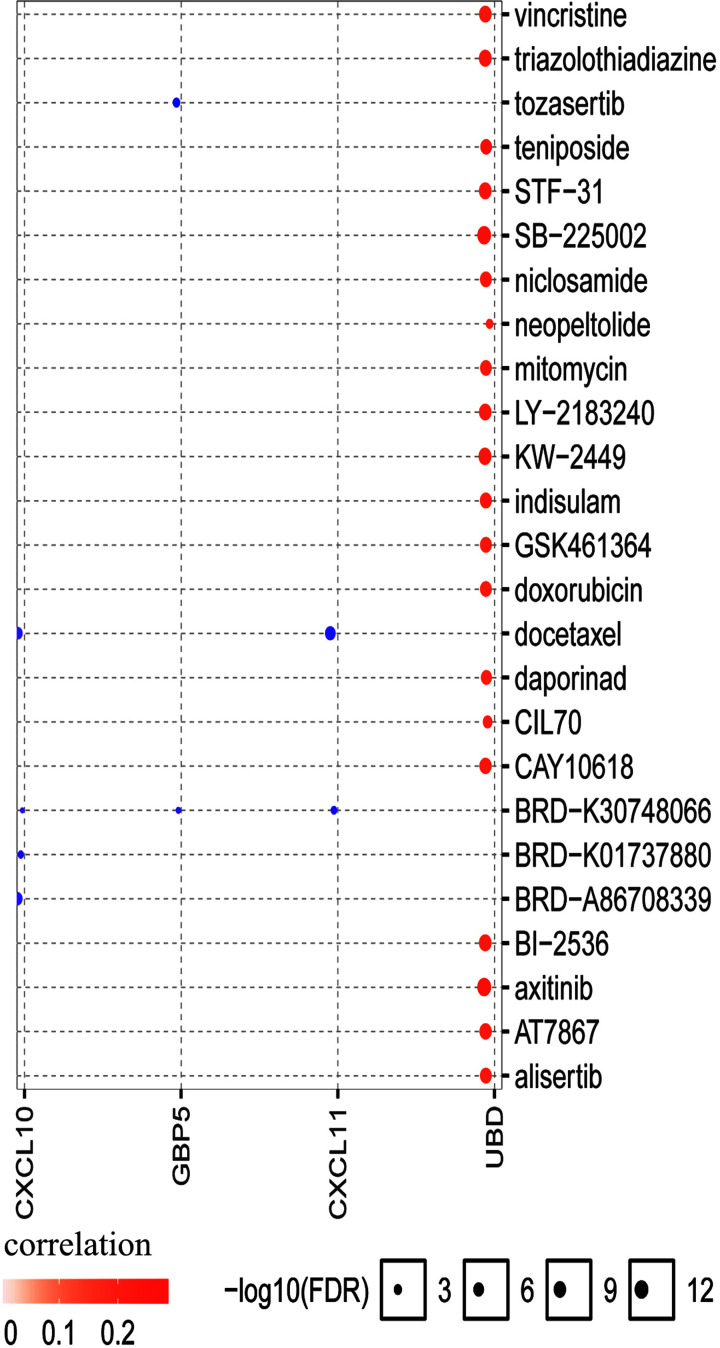
Drug resistance analysis of the central gene, CXCL10, using GSCALite

For drug sensitivity analysis, we assessed the correlation between CXCL10 expression and IC_50_ values of molecules from the Cancer Drug Sensitivity Genomics (GDSC) database. Our data showed that four drugs or small molecules were effective in association with reduced expression of CXCL10 (Figure 11). Specifically, CXCL10 was negatively regulated by docetaxel, BRD-K30748066, BRD-K01737880, and BRD-A86708339, supporting its utility as a potential therapeutic drug target for PTC.

## Discussion

PTC is a common malignant tumor of the endocrine system with increasing annual incidence. The tumor immune microenvironment is known to play a critical role in the evolution of malignancy and related to various biological behaviors of cancer, affecting tumor growth and spread. In the present study, we extracted data from the TCGA database to explore genes in the tumor microenvironment affecting overall survival, tumorigenesis and development of PTC. Furthermore, biomarkers related to prognosis and treatment in the microenvironment of PTC were identified. The relationships between clinicopathological variables and immune and stromal scores in PTC were initially evaluated. As shown in Figure 1, immune and stromal scores were related to tumor stage. With increase in the tumor staging level, the corresponding average immune and stromal scores as well as ESTIMATE score were increased, supporting the potential value of immune and stromal scores in the treatment and prediction of PTC. After grouping of all samples based on high and low immunity/stromal scores, 854 differentially expressed genes (DEGs) were identified. To clarify the roles of these DEGs in immune and stromal systems, we explored their functions in PTC. GO analysis disclosed the involvement of DEGs in leukocyte migration, leukocyte chemotaxis, cell chemotaxis, chemokine-mediated signaling pathways, response to chemokines, cellular response to chemokines and myeloid leukocyte migration. KEGG pathway analysis showed that DEGs are involved in cytokine–cytokine receptor interactions and the chemokine signaling pathway. Both GO and KEGG data suggest that the functions of these DEGs are closely related to specific pathways and molecular functions in the tumor microenvironment in PTC. Our results are in agreement with previous studies showing that aberrant expression of specific genes contributes significantly to development of the PTC tumor microenvironment through regulatory effects on the activities of various immune cells together with extracellular matrix molecules [32,33].

To further clarify the interactions between DEGs in PTC, we used STRING and Cytoscape software to construct protein–protein interaction networks and identify central genes. As shown in the results, the top five connected degree genes were CXCL10, CCL20, CCL5, CD4 and CXCL1. The top three PPI networks of CXCL10, KRTs, and ZAP70 modules were further selected as core modules. Immune evasion is a signature of tumorigenesis [34]. An earlier study by Marina et al. [35] reported that the gene encoding the chemokine CCL20 is overexpressed in PTC compared with normal thyroid tissue and independent of oncogene (RET/PTC or BRAF) status, suggesting the involvement of this chemokine in tumor-associated inflammation and the microenvironment. Several researchers have additionally demonstrated that four chemokines, CXCL1, CCL5, CCL20 and CCL21, biologically affect thyroid cancer cells by modulating inflammation and the immune microenvironment [36–40]. Abnormal secretion of CXCL10 in PTC and its involvement in regulation of malignant biological behavior have been previously reported [41,42]. Here, we further explored the association between DEGs and prognosis of PTC and identified 15 genes related to the tumor microenvironment. High expression of GATA5, LRRN4CL, OGDHL, PSAT1, SLC25A47, and TWIST2 was associated with poorer overall survival rates and high expression of CXCL10, ADGRG5, ASB2, DMBT1, GPR34, GZMK, GZMM, HTRA1 and TBX21with longer overall survival rates in thyroid carcinoma patients. Accumulating evidence suggests that aberrant expression of CXCL family peptides leads to abnormal recruitment of immune cells in tumors and various types of immune cells can increase CXCL peptide secretion by modulating the corresponding signaling pathways (particularly NF-kappaB), which suppress the immune surveillance status of the organism, ultimately resulting in a vicious cycle and reduced survival of cancer patients [43,44]. In view of the finding that the degree of connectivity of CXCL10 gene was up to 23 and associated with PTC survival, we selected CXCL10 as a core gene for analysis. Our data suggest that the CXCL10 gene plays an important regulatory role in the immune microenvironment of PTC, supporting its utility as a novel immunotherapeutic target. Next, we focused on the mechanisms underlying the function of CXCL10 in PTC. Immunochemical staining experiments revealed that CXCL10 was expressed at lower levels in PTC than paracancerous tissues. GSEA pathway enrichment analysis further showed low expression of CXCL10 in multiple cancer pathways (p53 signaling pathway, Cell cycle, Pathways in cancer, Colorectal cancer signal pathway).The LinkedOmics database was used to explore the top 50 positively and negatively correlated genes and kinase targets of CXCL10.As shown in Figure 5B,C, the top five genes negatively associated with CXCL10 were CXCL11, CXCL9, GBP5, UBD, and IL12RB1 and the top five genes positively associated with CXCL10 were CMTM4, TOM1L2, LCLAT1, CRY2, and PCBD2 with CXCL10. LCK, LYN, HCK, and SYK were the main kinase targets of CXCL10. All four kinases, along with CXCL10 were significantly involved in T-cell activation, regulation of lymphocyte activation, regulation of leukocyte activation, regulation of T-cell activation, regulation of cell activation, antigen receptor-mediated signaling and T-cell receptor signaling pathways. The kinase LCK belongs to the Src family and is critical for T-cell proliferation and activation in physiological conditions. Under pathological conditions, aberrantly expressed LCKs can directly trigger an inflammatory immune response [45]. In addition, LCK has been shown to be overexpressed in many cancer types and LCK inhibitors have been clinically used to treat a number of solid cancers [46–48]. Another study by Silvia and co-workers demonstrated that LCK kinases are involved in regulating the progression of thyroid cancer [49]. The collective results suggest that kinases play regulatory roles in the evolution of cancer. In many solid tumors, the degree of immune cell infiltration is strongly associated with patient prognosis [50,51]. In our experiments, Significant correlations were detected between CXCL10 and expression of immune markers, including PD-1 (PDCD1), CD2, CD19, CD79A, CD68, IL10, VSIG4, HLA-DPB1, CD11c (ITGAX), T-bet (TBX21), STAT1, IFN-g (IFNG), FOXP3 and CTLA4, suggesting that the CXCL10 gene exerts its effects in the thyroid cancer microenvironment by influencing immune cells and related molecules. Our results showed that CXCL10 is related to the overall survival of PTC patients and positively correlated with the infiltration level of B cells, CD8 + T cells, CD4 + T cells, macrophages, neutrophils and dendritic cells. While the CXCL10 gene could be used to provide additional information on the correlation between immune cell infiltration and clinical outcomes of PTC patients, the specific mechanisms of action of CXCL10 in PTC remain to be elucidated. The tumor microenvironment is composed of immune and stromal cells that participate in the progression of cancer and interact with PTC, thereby affecting occurrence, development, migration, metastasis and prognosis of cancer. The present study focused on the correlation between immune/stromal scores and PTC clinicopathological variables and explored the functions and prognostic value of genes related to the tumor microenvironment. We selected CXCL10 as a potential core gene of the PTC microenvironment and showed its significant correlation with prognosis and immune cells in PTC. CXCL10 expression was additionally regulated by a number of small-molecule drugs, including docetaxel, BRD-K30748066, BRD-K01737880, and BRD-A86708339. The collective evidence supports the utility of CXCL10 as a potential therapeutic drug target and novel potential biomarker for improving management and prognosis of PTC.

## Data Availability

The data used to support the findings of this study are available from the corresponding author upon request.

## Abbreviations

DEG: differentially expressed gene
IHC: immunohistochemical
PPI: protein–protein interaction
PTC: papillary thyroid cancer
TC: thyroid cancer

## References

[1] Jung K.W., Won Y.J., Kong H.J. et al. (2015) Cancer statistics in Korea: incidence, mortality, survival, and prevalence in 2012. Cancer Res. Treat. 47, 127–141 10.4143/crt.2015.060 25761484 PMC4398120

[2] Siegel R.L., Miller K.D. and Jemal A. (2019) Cancer statistics, 2019. CA Cancer J Clin. 69, 7–34 10.3322/caac.21551 30620402

[3] Zhu G., Deng Y., Pan L. et al. (2019) Clinical significance of the BRAFV600E mutation in PTC and its effect on radioiodine therapy. Endocr. Connect 8, 754–763 10.1530/EC-19-0045 31071680 PMC6547306

[4] Kim S.K., Woo J.W., Lee J.H. et al. (2016) Prophylactic Central Neck Dissection Might Not Be Necessary in Papillary Thyroid Carcinoma: Analysis of 11,569 Cases from a Single Institution. J. Am. Coll. Surg. 222, 853–864 10.1016/j.jamcollsurg.2016.02.001 27113516

[5] Ruiz Pardo J., Rodriguez J.M. and Rios A. (2020) Reply - Risk Factors of Metastatic Lymph Nodes in Papillary Thyroid Microcarcinoma. Cir. Esp. 98, 497–498 32622516 10.1016/j.ciresp.2020.06.001

[6] Zhou B., Wei L. and Qin J. (2020) The metastasis rate and high-risk factors for ipsilateral lateral neck lymph node metastasis in patients with papillary thyroid microcarcinoma (cT1aN0). Asian J. Surg. 43, 959–960 10.1016/j.asjsur.2020.05.018 32532680

[7] Tuttle R.M., Ball D.W., Byrd D. et al. (2010) Thyroid carcinoma. J. Natl. Compr. Canc. Netw. 8, 1228–1274 10.6004/jnccn.2010.0093 21081783

[8] Lango M., Flieder D., Arrangoiz R. et al. (2013) Extranodal extension of metastatic papillary thyroid carcinoma: correlation with biochemical endpoints, nodal persistence, and systemic disease progression. Thyroid 23, 1099–1105 10.1089/thy.2013.0027 23421588 PMC3770240

[9] Brown R.L., de Souza J.A. and Cohen E.E. (2011) Thyroid cancer: burden of illness and management of disease. J. Cancer 2, 193–199 10.7150/jca.2.193 21509149 PMC3079916

[10] Xing M., Liu R., Liu X. et al. (2014) BRAF V600E and TERT promoter mutations cooperatively identify the most aggressive papillary thyroid cancer with highest recurrence. J. Clin. Oncol. 32, 2718–2726 10.1200/JCO.2014.55.5094 25024077 PMC4145183

[11] Kim R., Emi M. and Tanabe K. (2007) Cancer immunoediting from immune surveillance to immune escape. Immunology 121, 1–14 10.1111/j.1365-2567.2007.02587.x 17386080 PMC2265921

[12] Gajewski T.F., Schreiber H. and Fu Y.X. (2013) Innate and adaptive immune cells in the tumor microenvironment. Nat. Immunol. 14, 1014–1022 10.1038/ni.2703 24048123 PMC4118725

[13] Romero-Garcia S., Moreno-Altamirano M.M., Prado-Garcia H. and Sanchez-Garcia F.J. (2016) Lactate Contribution to the Tumor Microenvironment: Mechanisms, Effects on Immune Cells and Therapeutic Relevance. Front. Immunol. 7, 52 26909082 10.3389/fimmu.2016.00052 PMC4754406

[14] Lambrechts D., Wauters E., Boeckx B. et al. (2018) Phenotype molding of stromal cells in the lung tumor microenvironment. Nat. Med. 24, 1277–1289 29988129 10.1038/s41591-018-0096-5

[15] Ferrari S.M., Fallahi P., Galdiero M.R. et al. (2019) Immune and Inflammatory Cells in Thyroid Cancer Microenvironment. Int. J. Mol. Sci. 20, 4413–4435 10.3390/ijms20184413 PMC6769504 31500315

[16] Gentles A.J., Newman A.M., Liu C.L. et al. (2015) The prognostic landscape of genes and infiltrating immune cells across human cancers. Nat. Med. 21, 938–945 10.1038/nm.3909 26193342 PMC4852857

[17] Gooden M.J., de Bock G.H., Leffers N., Daemen T. and Nijman H.W. (2011) The prognostic influence of tumour-infiltrating lymphocytes in cancer: a systematic review with meta-analysis. Br. J. Cancer 105, 93–103 10.1038/bjc.2011.189 21629244 PMC3137407

[18] Yoshihara K., Shahmoradgoli M., Martinez E. et al. (2013) Inferring tumour purity and stromal and immune cell admixture from expression data. Nat. Commun. 4, 2612 10.1038/ncomms3612 24113773 PMC3826632

[19] Priedigkeit N., Watters R.J., Lucas P.C. et al. (2017) Exome-capture RNA sequencing of decade-old breast cancers and matched decalcified bone metastases. JCI Insight 2, 10.1172/jci.insight.95703 28878133 PMC5621874

[20] Alonso M.H., Ausso S., Lopez-Doriga A. et al. (2017) Comprehensive analysis of copy number aberrations in microsatellite stable colon cancer in view of stromal component. Br. J. Cancer 117, 421–431 10.1038/bjc.2017.208 28683472 PMC5537504

[21] Ritchie M.E., Phipson B., Wu D. et al. (2015) limma powers differential expression analyses for RNA-sequencing and microarray studies. Nucleic Acids Res. 43, e47 10.1093/nar/gkv007 25605792 PMC4402510

[22] Li G.M., Zhang C.L., Rui R.P., Sun B. and Guo W. (2018) Bioinformatics analysis of common differential genes of coronary artery disease and ischemic cardiomyopathy. Eur. Rev. Med. Pharmacol. Sci. 22, 3553–3569 29917210 10.26355/eurrev_201806_15182

[23] Szklarczyk D., Gable A.L., Lyon D. et al. (2019) STRING v11: protein-protein association networks with increased coverage, supporting functional discovery in genome-wide experimental datasets. Nucleic Acids Res. 47, D607–D613 10.1093/nar/gky1131 30476243 PMC6323986

[24] Kanehisa M., Furumichi M., Tanabe M., Sato Y. and Morishima K. (2017) KEGG: new perspectives on genomes, pathways, diseases and drugs. Nucleic Acids Res. 45, D353–D361 10.1093/nar/gkw1092 27899662 PMC5210567

[25] Gene Ontology, C. (2006) The Gene Ontology (GO) project in 2006. Nucleic Acids Res. 34, D322–D326 10.1093/nar/gkj021 16381878 PMC1347384

[26] Vasaikar S.V., Straub P., Wang J. and Zhang B. (2018) LinkedOmics: analyzing multi-omics data within and across 32 cancer types. Nucleic Acids Res. 46, D956–D963 10.1093/nar/gkx1090 29136207 PMC5753188

[27] Li T., Fan J., Wang B. et al. (2017) TIMER: A Web Server for Comprehensive Analysis of Tumor-Infiltrating Immune Cells. Cancer Res. 77, e108–e110 10.1158/0008-5472.CAN-17-0307 29092952 PMC6042652

[28] Koscielny G., An P., Carvalho-Silva D. et al. (2017) Open Targets: a platform for therapeutic target identification and validation. Nucleic Acids Res. 45, D985–D994 10.1093/nar/gkw1055 27899665 PMC5210543

[29] Warde-Farley D., Donaldson S.L., Comes O. et al. (2010) The GeneMANIA prediction server: biological network integration for gene prioritization and predicting gene function. Nucleic Acids Res. 38, W214–W220 10.1093/nar/gkq537 20576703 PMC2896186

[30] Liu C.J., Hu F.F., Xia M.X., Han L., Zhang Q. and Guo A.Y. (2018) GSCALite: a web server for gene set cancer analysis. Bioinformatics 34, 3771–3772 10.1093/bioinformatics/bty411 29790900

[31] Remmele W. and Stegner H.E. (1987) Recommendation for uniform definition of an immunoreactive score (IRS) for immunohistochemical estrogen receptor detection (ER-ICA) in breast cancer tissue. Pathologe 8, 138–140 3303008

[32] Yu H., Huang X., Liu X. et al. (2013) Regulatory T cells and plasmacytoid dendritic cells contribute to the immune escape of papillary thyroid cancer coexisting with multinodular non-toxic goiter. Endocrine 44, 172–181 10.1007/s12020-012-9853-2 23264145

[33] Yin M., Di G. and Bian M. (2018) Dysfunction of natural killer cells mediated by PD-1 and Tim-3 pathway in anaplastic thyroid cancer. Int. Immunopharmacol. 64, 333–339 10.1016/j.intimp.2018.09.016 30243069

[34] Hanahan D. and Weinberg R.A. (2011) Hallmarks of cancer: the next generation. Cell 144, 646–674 21376230 10.1016/j.cell.2011.02.013

[35] Muzza M., Degl'Innocenti D., Colombo C. et al. (2010) The tight relationship between papillary thyroid cancer, autoimmunity and inflammation: clinical and molecular studies. Clin. Endocrinol. (Oxf) 72, 702–708 10.1111/j.1365-2265.2009.03699.x 20447069

[36] Rotondi M., Coperchini F., Latrofa F. and Chiovato L. (2018) Role of Chemokines in Thyroid Cancer Microenvironment: Is CXCL8 the Main Player? Front. Endocrinol. (Lausanne) 9, 314 10.3389/fendo.2018.00314 29977225 PMC6021500

[37] Melillo R.M., Guarino V., Avilla E. et al. (2010) Mast cells have a protumorigenic role in human thyroid cancer. Oncogene 29, 6203–6215 10.1038/onc.2010.348 20729915

[38] Zeng W., Chang H., Ma M. and Li Y. (2014) CCL20/CCR6 promotes the invasion and migration of thyroid cancer cells via NF-kappa B signaling-induced MMP-3 production. Exp. Mol. Pathol. 97, 184–190 10.1016/j.yexmp.2014.06.012 24984269

[39] Zhang Y.Y., Liu Z.B., Ye X.G. and Ren W.M. (2016) Iodine regulates G2/M progression induced by CCL21/CCR7 interaction in primary cultures of papillary thyroid cancer cells with RET/PTC expression. Mol. Med. Rep. 14, 3941–3946 10.3892/mmr.2016.5686 27574129

[40] Kwon K.H., Lee Y.C., Chung J.H. and Eun Y.G. (2013) Association study of chemokine (C-C motif) ligand 5 gene polymorphism and papillary thyroid cancer. J. Invest. Surg. 26, 319–324 10.3109/08941939.2013.805857 23957698

[41] Antonelli A., Ferrari S.M., Fallahi P. et al. (2009) Dysregulation of secretion of CXC alpha-chemokine CXCL10 in papillary thyroid cancer: modulation by peroxisome proliferator-activated receptor-gamma agonists. Endocr. Relat. Cancer 16, 1299–1311 10.1677/ERC-08-0337 19755523

[42] Fallahi P., Ferrari S.M., Piaggi S. et al. (2018) The paramount role of cytokines and chemokines in papillary thyroid cancer: a review and experimental results. Immunol. Res. 66, 710–722 10.1007/s12026-018-9056-x 30617967

[43] Susek K.H., Karvouni M., Alici E. and Lundqvist A. (2018) The Role of CXC Chemokine Receptors 1-4 on Immune Cells in the Tumor Microenvironment. Front. Immunol. 9, 2159 10.3389/fimmu.2018.02159 30319622 PMC6167945

[44] Taki M., Abiko K., Baba T. et al. (2018) Snail promotes ovarian cancer progression by recruiting myeloid-derived suppressor cells via CXCR2 ligand upregulation. Nat. Commun. 9, 1685 10.1038/s41467-018-03966-7 29703902 PMC5923228

[45] Cancer Genome Atlas, N. (2015) Genomic Classification of Cutaneous Melanoma. Cell 161, 1681–1696 10.1016/j.cell.2015.05.044 26091043 PMC4580370

[46] Santpere G., Alcaraz-Sanabria A., Corrales-Sanchez V., Pandiella A., Gyorffy B. and Ocana A. (2018) Transcriptome evolution from breast epithelial cells to basal-like tumors. Oncotarget 9, 453–463 10.18632/oncotarget.23065 29416627 PMC5787480

[47] Clarke C.N., Lee M.S., Wei W. et al. (2017) Proteomic Features of Colorectal Cancer Identify Tumor Subtypes Independent of Oncogenic Mutations and Independently Predict Relapse-Free Survival. Ann. Surg. Oncol. 24, 4051–4058 10.1245/s10434-017-6054-5 28936799 PMC6063735

[48] Chen R. and Chen B. (2015) The role of dasatinib in the management of chronic myeloid leukemia. Drug Des. Devel. Ther. 9, 773–779 10.2147/DDDT.S80207 25709401 PMC4330036

[49] Ferrari S.M., La Motta C., Sartini S. et al. (2016) Pyrazolopyrimidine Derivatives as Antineoplastic Agents: with a Special Focus on Thyroid Cancer. Mini Rev. Med. Chem. 16, 86–93 10.2174/1389557515666151016124208 26471970

[50] Ino Y., Yamazaki-Itoh R., Shimada K. et al. (2013) Immune cell infiltration as an indicator of the immune microenvironment of pancreatic cancer. Br. J. Cancer 108, 914–923 10.1038/bjc.2013.32 23385730 PMC3590668

[51] Schneider K., Marbaix E., Bouzin C. et al. (2018) Immune cell infiltration in head and neck squamous cell carcinoma and patient outcome: a retrospective study. Acta Oncol. 57, 1165–1172 10.1080/0284186X.2018.1445287 29493423

